# Flipped classroom-based application of Peyton’s four-step approach in standardized training of ultrasound residents for thyroid and cervical lymph node zoning

**DOI:** 10.7717/peerj.18633

**Published:** 2024-12-18

**Authors:** Jiajia Wang, Yunyun Zhan, Biyun Sun, Yu Bi, Rubing Li, Fan Jiang, Mei Peng

**Affiliations:** 1 Department of Ultrasound Medicine, the Second Affiliated Hospital of Anhui Medical University, Hefei, China; 2 Department of Ultrasound Medicine of the first Affiliated Hospital of Wannan Medical College, Wuhu, Anhui, China

**Keywords:** Ultrasound, Medical education, Teaching method, Thyroid

## Abstract

**Background:**

To investigate whether combining the flipped classroom approach with Peyton’s four-step method can enhance teaching effectiveness in ultrasound (US) zoning of the thyroid and cervical lymph nodes for standardized residency training.

**Methods:**

A total of 66 resident training students were randomly divided into a control group and an observation group. The control group received traditional teaching methods, including “see one, do one” learning, lecture-based learning (LBL), and case-based learning (CBL). The observation group was taught using Peyton’s four-step teaching method, the flipped classroom approach, and CBL. Assessments were conducted through skill operation and clinical case analysis. A questionnaire survey was used to evaluate student satisfaction. Assessment scores and questionnaire ratings between the two teaching methods were compared.

**Results:**

1) Assessment results demonstrated higher scores in skill operation and clinical case analysis for the observation group compared to the control group [(87.64 ± 3.72) *vs*. (80.48 ± 5.92) points, (87.94 ± 4.46) *vs*. (82.85 ± 4.24) points]. 2) The questionnaire survey indicated that resident trainees taught using Peyton’s four-step method showed greater improvement in learning interest [(4.61 ± 0.57) *vs*. (3.70 ± 0.85) points] and experienced reduced exam pressure compared to the control group [(4.52 ± 0.62) *vs*. (3.21 ± 0.70) points]. These differences were statistically significant (*P* < 0.05).

**Conclusion:**

Peyton’s four-step approach combined with the flipped classroom method improved resident training students’ scores in skill operation and clinical case analysis for ultrasound zoning of the thyroid and cervical lymph nodes. It also stimulated learning interest and alleviated exam pressure, making it an effective teaching method for enhancing standardized US training outcomes in resident education.

## Introduction

Standardized residency training is essential for medical students to become qualified resident physicians. The Chinese standardized residency training program was initiated in 2014 and has evolved over the past decade to align with the clinical medical environment in China (Zhang et al., 2024). Ultrasound medicine, a clinical medical imaging course reliant on hands-on skills and case analysis, requiring resident students to have both skill operation and case analysis abilities (Bahner et al., 2014). The evaluation of thyroid and cervical lymph node ultrasound is pivotal within ultrasound residency training and has consistently received emphasis. Particularly, with the development of medicine, there is a growing emphasis on active surveillance of papillary thyroid carcinoma (Song, Kim & Kang, 2022). This means that higher requirements are put forward for ultrasound practitioners in the assessment of thyroid and cervical lymph nodes.

Although artificial intelligence or deep learning-based classification algorithms can be utilized in clinical practice for the differential diagnosis of benign and malignant thyroid and cervical lymph node ultrasound images, thereby assisting clinicians in enhancing the accuracy of disease diagnosis, it remains imperative for residents-in-training to acquire proficiency in ultrasound skills in thyroid and cervical lymph node as well as disease identification. Previous study indicates that the Chinese-Thyroid Image Reporting And Data System (C-TIRADS) classification can reduce the doctors’ subjective dependence in the diagnosis of thyroid nodules and improve the accuracy of diagnosing benign and malignant thyroid nodules, and C-TIRADS classification is the standard for evaluating thyroid nodules in ultrasound resident examination currently (Miao et al., 2020). A review of previous ultrasound resident students revealed several educational challenges were identified in the examination and diagnosis of thyroid and cervical lymph nodes: 1) Incorrect adjustment of instruments during thyroid color doppler examination; 2) Insufficient proficiency in interpreting thyroid nodules using C-TIRADS classification; 3) Difficulty in recognizing anatomical structures within cervical lymph node zones on ultrasound images. Therefore, it is necessary to find a feasible teaching method to address these challenges, aiming to enhance resident trainees’ abilities in cervical ultrasound operation, improve their skills in interpreting ultrasound images, and refine their case analysis capabilities to enhance overall teaching effectiveness.

The elements of episodic memory have been used in teaching for 40 years, and it is believed that they can help students generate new perspectives and insight, but they lack application experience in the field of medical education (Martin-Ordas & Easton, 2024). The Peyton’s four-step teaching method, renowned for its comprehensive approach to skill acquisition and retention in various medical fields, has gained recognition due to its integration of demonstration, deconstruction, comprehension, and performance (Rossettini et al., 2017). Peyton’s four-step consists of four stages: demonstration, deconstruction, comprehension, and performance (Giacomino, Caliesch & Sattelmayer, 2020). It has primarily been applied in clinical medical skills training and has achieved positive teaching outcomes. Since the teaching process of Peyton’s four-step approach is similar to the path of human cognitive learning, especially in the “comprehension” stage, where students actively participate in the learning process, it allows self-assessment of students through evaluating “how to guide others” and assessing the effectiveness of their guidance. Thus, Peyton’s four-step approach is recognized as effective in teaching clinical skills. However, its application in ultrasound skills education remains relatively limited, suggesting the necessity of further exploration and validation of its value in this specialized domain (Gradl-Dietsch et al., 2018).

Compared to traditional teaching models, the flipped classroom approach is different in both structure and instructional design (Liu et al., 2024). It is student-centered promoting independent learning. Teachers provide slides and videos to resident trainees through online platforms prior to class, allowing them to organize their study schedules and acquire pre-class knowledge independently. In-class time is then focused on targeted learning, addressing specific student needs. After class, resident trainees can revisit the materials, reinforcing their understanding, particularly in challenging areas. This model allows flexibility in terms of time and location, creating a personalized learning experience (Canellas & Kendall, 2018).

Given the previous challenges in teaching thyroid and cervical lymph node ultrasound, we posed a hypothesis that Peyton’s four-step approach combined flipped classroom model can improved the theident trainees’ performance in performing ultrasound operations, read ultrasound images, and analyzing clinical ultrasound cases, providing a reference for ultrasound standardized training teaching methods. This study aimed to explore whether the new teaching model can improve the ultrasound teaching effect.

## Materials and Methods

### Participants

In this study, all procedures performed in this study were approved by the ethics committee of the Second Affiliated Hospital of Anhui Medical University and obtained ethics approval in written (SL-YX2024-052).

**Figure 1:**
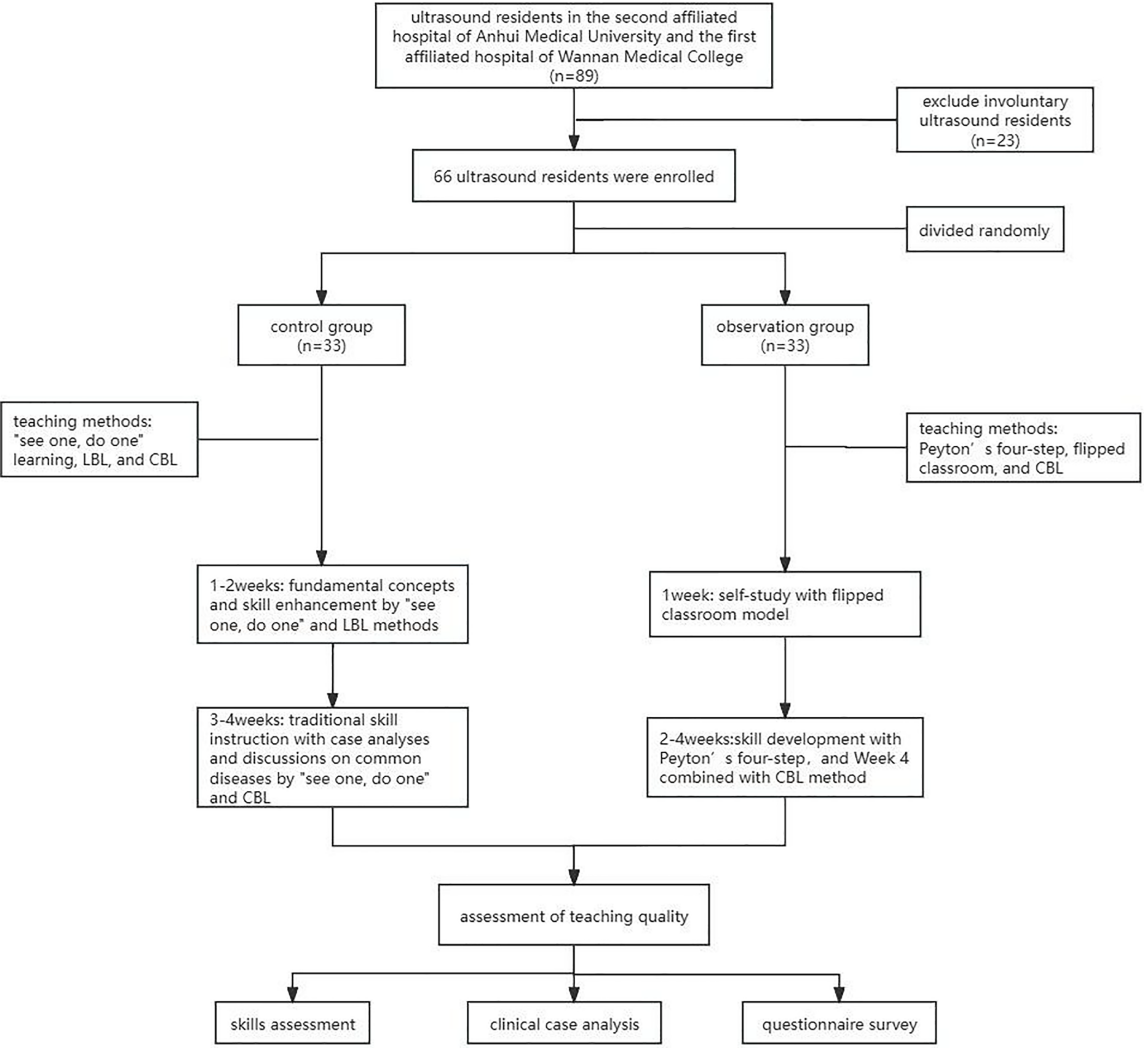
The flow chart of training process. LBL, lecture-based learning; CBL, case-based learning.

A total of 89 students from the Department of Ultrasound Medicine in the Second Affiliated Hospital of Anhui Medical University and the First Affiliated Hospital of Wannan Medical College were initially enrolled in the study. These 66 resident trainees were randomly divided into two groups: a control group and an observation group, each consisting of 33 resident trainees. The control group included 13 males and 22 females, aged 23 to 36 years, with an average age of (26.61 ± 2.73) years. The observation group comprised eight males and 25 females, aged 23 to 30 years, with an average age of (26.18 ± 1.61) years. There was no statistically significant difference in age between the two groups. All participants in this study consent this study, and written informed consent. The detail of this research process was demonstrated in Fig. 1.

### Teaching groups

#### Control group

The control group students were instructed using traditional teaching methods, which including “see one, do one” learning, lecture-based learning (LBL), and case-based learning (CBL) (He et al., 2024).

**“**See one, do one**”** learning involved the teacher’s demonstration of ultrasound skills, standardized teaching of ultrasound section examination, explanation of key points in skill operation, followed by students performing ultrasound operations.

LBL referred to traditional classroom teaching, where the instructor delivered PowerPoint (PPT) lectures on educational content aligned with the learning objectives.

CBL referred to the case-based teaching method where the instructor explains common clinical diseases related to the educational goals through case presentations during the classroom teaching process.

#### Observation group

The observation group students were taught by Peyton’s four-step approach, flipped classroom, and CBL (Avakyan & Taylor, 2024).

Peyton’s four-step approach included structured stages for teaching ultrasound skills focused on thyroid and cervical lymph nodes: (1) Demonstration: The instructor performed ultrasound scans of the thyroid and cervical lymph nodes, focusing on scanning techniques without theoretical explanations. (2) Deconstruction: During repeated ultrasound scans, the instructor simultaneously lectured on ultrasound scanning techniques, normal sonographic appearances of the thyroid, anatomical localization of cervical lymph node zones, and recognition of ultrasound images. Common diseases associated with abnormal lymph nodes in various regions were also discussed. (3) Comprehension: Resident trainees repeated the lecture content provided by the instructor. The instructor performed a third ultrasound scan of the thyroid and cervical lymph node zones based on the trainees’ recollection, and corrected any errors or omissions during the scanning process. (4) Performance: Resident trainees independently conducted ultrasound scans of the thyroid and cervical lymph node zones, concentrating on scanning techniques, anatomical positioning, and ultrasound image interpretation.

### Teaching methods

#### Formation of the teaching team

The teaching team consisted of a teaching director, eight instructors (including teaching teacher, skill assessment teacher, clinical case assessment teacher and clinical case presentation teacher), and a teaching secretary. The teaching director, with over 20 years of experience in ultrasound clinical practice and clinical teaching, was responsible for setting educational objectives, guiding teaching programs, and supervising the entire teaching process. Each instructor had more than 10 years of clinical experience in ultrasound and clinical teaching, contributing to the formulation of teaching programs and conducting teaching activities. Skill and clinical case assessment teachers, and clinical case assessment presentation teachers, hold senior professional titles in medicine and have over 5 years of experience in clinical ultrasound work.

Before starting the teaching activities, the instructors assigned to the observation group completed at least three consecutive months of training in Peyton’s four-step approach for US teaching.

#### Establishment of teaching objectives

The teaching objectives were developed based on the Clinical Practice Competency Assessment Standards for Ultrasound Medicine Specialty in the Chinese Standardized Residency Training (2022 version) (Zhou et al., 2024). The focus was on the following key competencies: (1) Proficiency in conducting comprehensive examinations and performing standardized measurements of the left and right lobes and isthmus of the thyroid, along with thorough examination of cervical lymph node zones with accurate ultrasound anatomical identification; (2) Ability to accurately describe ultrasound images of common thyroid and cervical lymph node lesions using standardized terminology, and to perform comprehensive and rational analysis of these lesions based on patient disease history and ultrasound features; (3) Competence in adjusting two-dimensional (2D) ultrasound and doppler ultrasound images using ultrasound equipment; (4) Capability to perform examinations of the superior thyroid artery and measure blood flow velocity and resistance index (RI) accurately; (5) Understanding of the latest advancements in imaging technologies applicable to common thyroid and cervical lymph node lesions.

#### Teaching plans for control and observation groups

The teaching of thyroid and cervical lymph node ultrasound spanned 4 weeks.

In the control group, weeks 1 and 2 utilized the “see one, do one” and LBL methods, focusing on fundamental concepts and skill enhancement in thyroid and cervical lymph node assessment. Weeks 3 and 4 continued the “see one, do one” approach while incorporating CBL. This phase combined traditional skill instruction with case analyses and discussions on common thyroid and cervical lymph node diseases.

For the observation group, week 1 involved self-study using course videos and PPT accessed through an online platform in a flipped classroom model. During this phase, students posed questions to teachers regarding challenging aspects encountered in their independent learning. Weeks 2, 3, and 4 focused around Peyton’s four-step approach, including demonstration, deconstruction, comprehension, and execution to enhance skill development. Week 4 emphasized the CBL method, with instructors leading classroom sessions to address student inquiries pertaining to both self-study and skill acquisition processes.

#### Assessment of teaching project

The teaching project assessment was conducted in the fourth week of instruction, including three parts: skills assessment, clinical case analysis, and a questionnaire survey (Zhang et al., 2024).

**Skills assessment:** Resident trainees were required to complete a comprehensive scan of the thyroid gland and lymph nodes within 5 min and retain seven images. The assessment was scored out of 100 points, with detailed scoring criteria as follows: (1) Score for the integrity of the saved ultrasound pictures (10 points); (2) Skilled adjustment of ultrasound instruments (10 points); (3) seven ultrasound images saved: thyroid transverse 2D image (10 points), left lobe longitudinal 2D image (10 points), left lobe longitudinal color doppler image (10 points), superior thyroid artery color doppler image (10 points), superior thyroid artery spectral doppler image (10 points), transverse section of the omohyoid muscle crossing the internal jugular vein (10 points), color doppler of lymph node (10 points); (4) Completion time (10 points): within 5 min (10 points), 5–8 min (5 points), over 8 min (0 points).

**Clinical case analysis:** Resident trainees were assessed through case studies, with a total of five questions worth 20 points each, leading to a maximum score of 100 points. The detailed scoring criteria were as follows: (1) Ability to summarize patients’ history and other clinical examinations (5 points); (2) Standardized description of ultrasound imaging features of the disease requiring C-TIRADS grading for thyroid lesions (10 points); (3) Disease diagnosis and differential diagnosis (5 points).

**Questionnaire survey:** After the assessment was completed, students completed a questionnaire survey comprising three items, showed in Table 1. Each item was worth five points (using the Likert five-point scale for scoring, with 1–5 points representing very dissatisfied, dissatisfied, basically satisfied, satisfied, and very satisfied, respectively). The total score for the questionnaire was 20 points.

**Table 1:** Teaching effectiveness satisfaction questionnaire indicators.

Assessment item content	Score (1–5 points)
Course’s ability to stimulate learning interest	
Course’s effectiveness in alleviating residency examination pressure	
Overall course satisfaction evaluation	

#### Project implementation and supervision

The teaching process was implemented by the teaching team in accordance with the teaching plan, which was developed based on the educational objectives and included assessment activities. The entire teaching process was supervised by the teaching director, who was also responsible for interpreting the results.

### Statistical analysis

SPSS 26.0 software (IBM, Armonk, NY, USA) was used for data analysis, skills assessment scores, clinical case analysis scores, and questionnaire survey scores were quantitative data, which were represented by mean ± standard deviation when they were aligned with normal distribution. Independent *t* test was used to analyze the test scores of the control group and the observation group. *P* < 0.05 was considered statistically significant.

## Results

### The participants information

We divided 66 students to control group (*n* = 33), and observation group (*n* = 33) randomly. The control group consisted of 13 male and 22 female students, aged between 23 and 36 years, with an average age of (26.61 ± 2.73) years. The observation group included 8 male and 25 female students, aged between 23 and 30 years, with an average age of (26.18 ± 1.61) years. There was no statistically significant difference in age between the two groups.

### Skill assessment and clinical case examination scores

**Figure 2:**
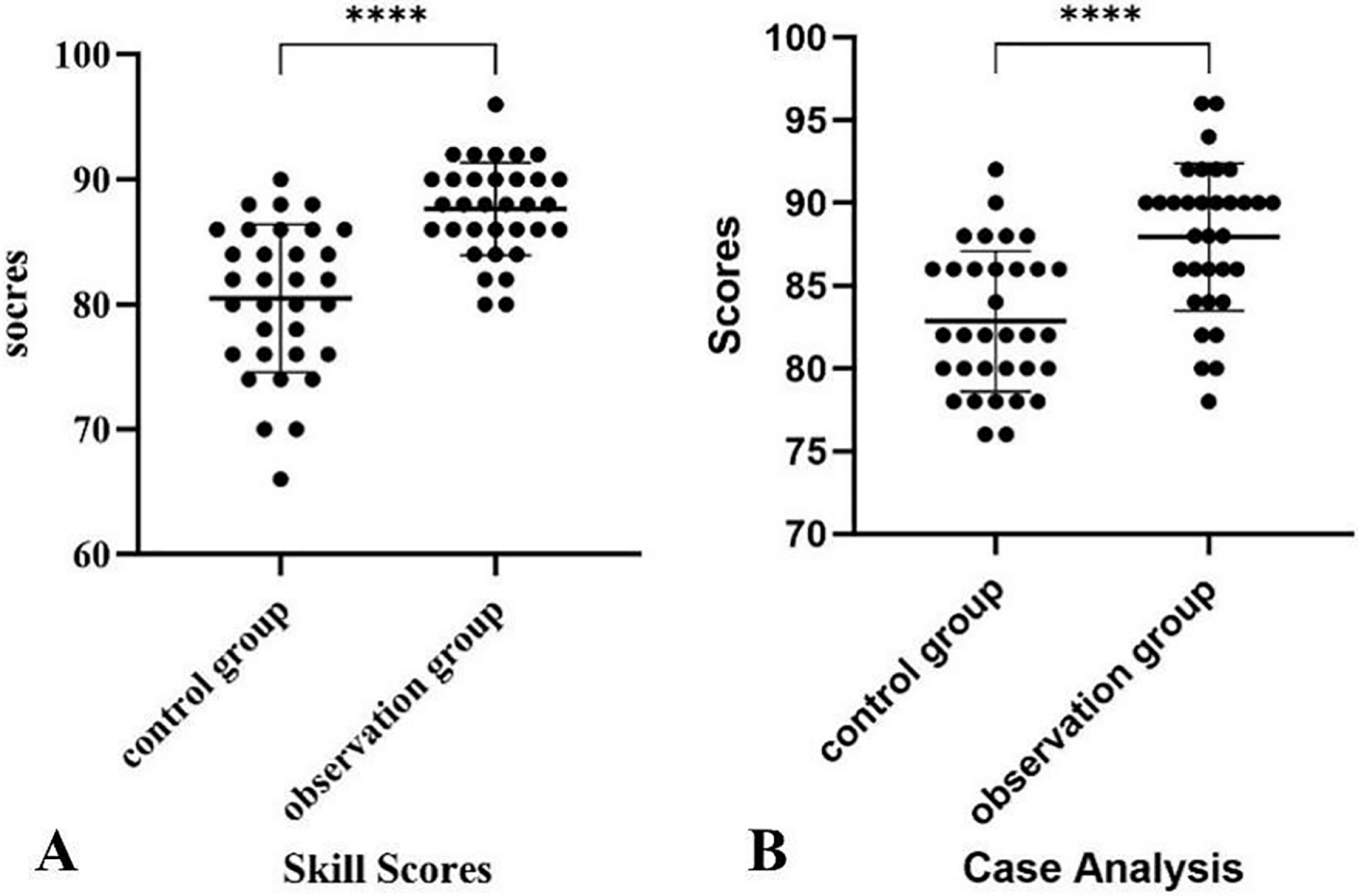
The assessment points results of the two groups of students. (A) The points of skills assessment (B) The points of clinical case analysis. The control group: *n* = 33; the observation group: *n* = 33. ****P < 0.001, denotes statistical significance.

The resident trainees in the observation group achieved higher scores in both skill operation and case analysis compared to those in the control group. Detailed results of the analysis can be found in Table 2 and Fig. 2. The differences between the two groups were statistically significant.

**Table 2:** The assessment points results of the two groups of students.

	Control group	Observation group	*t*	*P*
Skills assessment (Points)	80.48 ± 5.92	87.64 ± 3.72	5.877	<0.001
Clinical case analysis (Points)	82.85 ± 4.24	87.94 ± 4.46	4.752	<0.001

### Questionnaire survey ratings

In the satisfaction survey questionnaire, resident students in the observation group reported higher levels of satisfaction regarding their interest in learning and alleviated examination pressure compared to those in the control group, with this difference being statistically significant. However, there was no statistically significant difference was observed in overall teaching satisfaction between the two groups (Table 3).

**Table 3:** Questionnaire survey results of the two groups of students.

	Control group	Observation group	*t*	*P*
Course’s ability to stimulate learning interest (Points)	3.70 ± 0.85	4.61 ± 0.57	5.154	<0.001
Course’s effectiveness in alleviating residency examination pressure (Points)	3.21 ± 0.70	4.52 ± 0.62	8.037	<0.001
Overall course satisfaction evaluation (Points)	4.39 ± 0.56	4.42 ± 0.56	0.221	0.827

## Discussion

Compared to other medical specialties, standardized residency training in ultrasound medicine requires a balance between proficiency in ultrasound skill operation and the ability to analyze case images (Wakefield et al., 2018). In recent years, ongoing discussions regarding ultrasound residency training methods have led to continuous updates and improvements. This study demonstrated that integrating Peyton’s four-step approach, a flipped classroom model, and CBL enhances the performance of resident trainees in both skill operation and case image analysis. Simultaneously, it increased resident trainees’ interest in learning and reduced the psychological pressure associated with completing residency examinations.

Previous research on ultrasound teaching has demonstrated that the flipped classroom model can effectively address the limitations of traditional teaching methods in both ultrasound and non-ultrasound specialties, which are often constrained by classroom location and time restrictions (Skrzypek et al., 2019; Weimer et al., 2024). By providing students with learning materials and key points through online platforms before class, the flipped classroom enables students to prepare knowledge in advance, enhances student-instructor interaction during the learning process, and promotes students’ learning initiative (Moraros et al., 2015). In this study, for the observation group of resident trainees, traditional lectures were replaced with self-study activities during the first week. Subsequently, we observed that students in the observation group posed significantly more questions during in-person teaching sessions. The questionnaire survey further indicated that the observation group exhibited improved learning interest compared to the control group.

Previous discussions on teaching methods have predominantly focused on theoretical classroom instruction and examinations, with limited exploration of skill-teaching methodologies specific to ultrasound specialties. Peyton’s four-step approach comprises demonstration, deconstruction, comprehension, and performance, originally designed for teaching clinical skills (Krautter et al., 2015). Research indicates that this approach is similar to human memory processes and enhances learning compared to traditional methods by incorporating a “comprehension” step. It differs from the traditional “see one, do one” approach and allows students to independently absorb and understand skill instruction content, progressing through guided teaching from instructors. Peyton’s approach systematically integrates skill operation and advancement, integrating theoretical knowledge, practical application, and clinical critical thinking skills. Therefore, combining CBL with Peyton’s four-step approach facilitates the development of enhanced clinical critical thinking abilities in case analysis (Nourkami-Tutdibi et al., 2020). Therefore, this study showed that combining the Peyton’s four-step teaching and flipped classroom, not only can improve students’ examination scores, but more importantly, their clinical critical thinking abilities can be enhanced, which is an important part of a doctor’s clinical work and fills the limitations of traditional teaching methods in cultivating clinical reasoning ability.

Thyroid and cervical lymph nodes, as crucial components of the superficial ultrasound examination station in ultrasound residency training, have consistently been focal points in ultrasound teaching (Lin et al., 2024). This teaching design is based on experienced instructors specialized in superficial ultrasound, supervised by a teaching director with extensive clinical and educational expertise, ensuring consistency and rigor in teaching standards. Previous teaching experiences have highlighted significant psychological pressure among students during residency completion examinations, which are conducted face-to-face with examiners (Shafqat et al., 2022). These emotions often impair normal performance, directly impacting skill assessment and case analysis scores. Alleviating residents’ adverse psychological states hinges on enhancing their theoretical knowledge and skill proficiency (Mehta et al., 2013; Boccatonda et al., 2023). This study shows that the observation group students got higher points on the survey of alleviating examination pressurestress (4.52 ± 0.62) points *vs*. control group (3.21 ± 0.70) points, and the teachers noticed that after the students’ exam stress was alleviated, they were able to demonstrate the ultrasound examinating images more proficiently during the skill assessmet.

Furthermore, in the skill assessment process,in addition to capturing relatively simple thyroid ultrasound image capture, resident trainees were also required to perform ultrasound imaging of cervical lymph node anatomical structures. While this added complexity to the evaluation, it closely mirrors clinical practice demands. Implementing of Peyton’s four-step approach enabled students in the observation group to gain a more precise understanding of challenging anatomical localizations (Afzali et al., 2017). This improved their confidence in clinical operations by enhancing their comprehension of critical and complex aspects, thereby mitigating examination-related pressure and indirectly boosting their assessment scores.

This teaching study had several limitations. Firstly, it focused exclusively on analyzing thyroid and cervical lymph node ultrasound examinations within the superficial examination station of the residency completion test, omitting content from abdominal or cardiac examination stations. Whether the combination of the flipped classroom and Peyton’s four-step approach can demonstrate advantages in ultrasound residency training for other subspecialties requires further investigation. Secondly, our study was conducted only in two teaching hospitals in China, thus further research and exploration involving a broader range of teaching hospitals is necessary to determine the generalizability of the discussed flipped classroom and Peyton’s four-step method. Additionally, future teaching studies should encompass more training bases and include a larger cohort of resident trainees to broaden the scope of research and conduct more comprehensive educational investigations.

## Conclusions

In conclusion, this study implemented a teaching research project that integrated the flipped classroom with Peyton’s four-step approach in ultrasound residency training for thyroid and cervical lymph nodes. Results indicated that compared to traditional teaching methods, this combined approach effectively enhanced ultrasound residents’ skill scores and performance in case analysis. Furthermore, it fostered students’ interest in autonomous learning and alleviated examination pressure. This methodology holds promise for application in future ultrasound residency training to enhance teaching efficiency and quality.

## Supplemental Information

10.7717/peerj.18633/supp-1 Supplemental Information 1 Raw data. The observation group got higher scores in skill operation and clinical case analysis than higher scores in skill operation and clinical case analysis. The questionnaire survey results indicated that observation students can improved learning interest and reduced exam pressure compared to the control group.

10.7717/peerj.18633/supp-2 Supplemental Information 2 CONSORT.

10.7717/peerj.18633/supp-3 Supplemental Information 3 Questionnaire (Chinese).

10.7717/peerj.18633/supp-4 Supplemental Information 4 Questionnaire (English).

## Abbreviations

LBL: lecture-based learning
CBL: case-based learning
C-TIRADS: Chinese-Thyroid Image Reporting And Data System
PPT: PowerPoint
2D: two-dimensional
RI: resistance index

## Additional Information and Declarations

### Data Availability

The following information was supplied regarding data availability: The raw measurements are available in the Supplemental Files.
