# Supplementary figures and images for: Flipped classroom-based application of Peyton’s four-step approach in standardized training of ultrasound residents for thyroid and cervical lymph node zoning
Source: PeerJ. 2024 Dec 18;12:e18633. doi: 10.7717/peerj.18633 (PMC11662902; doi:10.7717/peerj.18633)

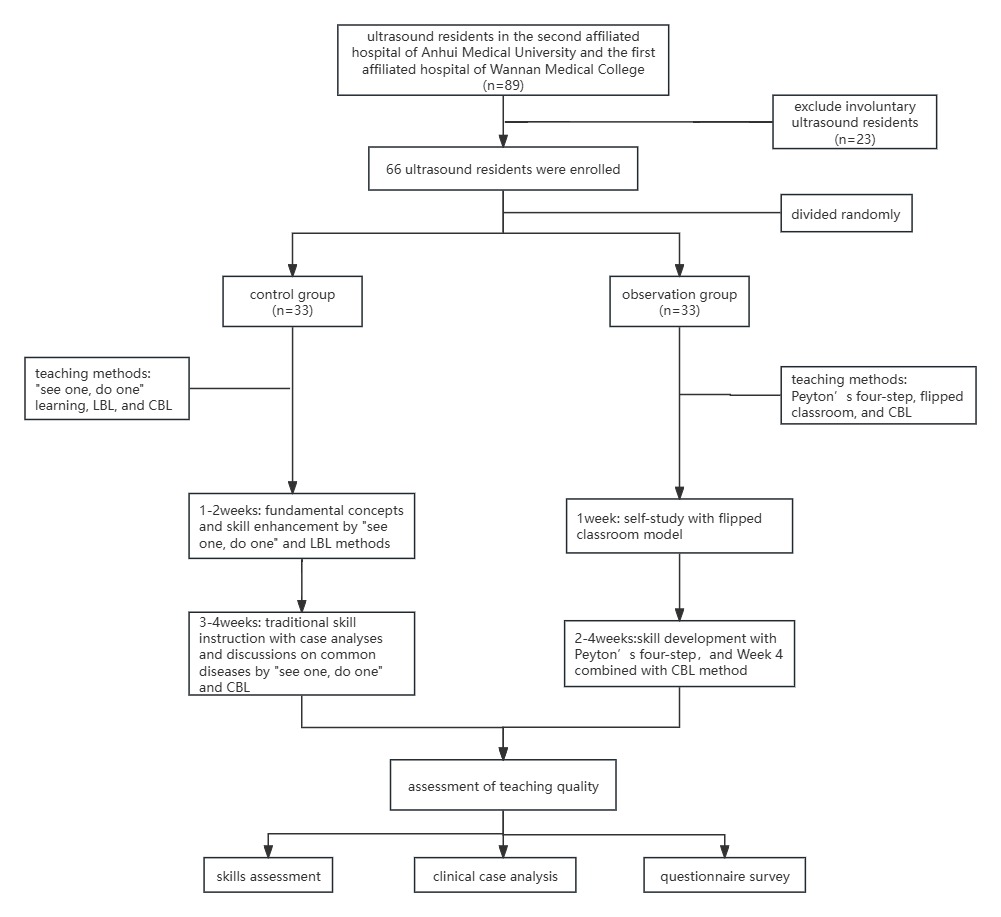

Supplement: Supplemental Information 2 [file peerj-12-18633-s002.jpg]
