# Supplementary material for: Flipped classroom-based application of Peyton’s four-step approach in standardized training of ultrasound residents for thyroid and cervical lymph node zoning
Source: PeerJ. 2024 Dec 18;12:e18633. doi: 10.7717/peerj.18633 (PMC11662902; doi:10.7717/peerj.18633)
Supplement: Supplemental Information 3 [file peerj-12-18633-s003.docx]

**问卷调查**

**教学效果满意度调查表**

| 条目 | 分数 (1-5 分) |
| --- | --- |
| 课程能否提高您的学习兴趣 |  |
| 课程能否有效缓解您的考试压力 |  |
| 总体课程满意度 |  |
