# Supplementary material for: Flipped classroom-based application of Peyton’s four-step approach in standardized training of ultrasound residents for thyroid and cervical lymph node zoning
Source: PeerJ. 2024 Dec 18;12:e18633. doi: 10.7717/peerj.18633 (PMC11662902; doi:10.7717/peerj.18633)
Supplement: Supplemental Information 4 [file peerj-12-18633-s004.docx]

**Questionnaire**

**Teaching Effectiveness Satisfaction Questionnaire Indicators**

| Assessment Item Content | Score (1-5 points) |
| --- | --- |
| Course’s ability to stimulate learning interest |  |
| Course’s effectiveness in alleviating residency examination pressure |  |
| Overall course satisfaction evaluation |  |
